# Home Blood Pressure Monitoring for Hypertension Diagnosis by Current Recommendations: A Long Way to Go

**DOI:** 10.1161/HYPERTENSIONAHA.121.18463

**Published:** 2021-12-02

**Authors:** Kelsey B. Bryant, Matthew B. Green, Daichi Shimbo, Joseph E. Schwartz, Ian M. Kronish, Yiyi Zhang, James P. Sheppard, Richard J. McManus, Andrew E. Moran, Brandon K. Bellows

**Affiliations:** Division of General Medicine, Icahn School of Medicine at Mount Sinai, NY (K.B.B.).; Division of General Medicine, Columbia Irving Medical Center, NY (M.B.G., D.S., J.E.S., I.M.K., Y.Z., A.E.M., B.K.B.).; Stony Brook University, NY (J.E.S.).; University of Oxford, United Kingdom (J.P.S., R.J.M.).

**Keywords:** blood pressure, body mass index, health insurance, hypertension, insurance coverage

Out-of-office blood pressure (BP) monitoring (eg, home BP monitoring [HBPM] or ambulatory BP monitoring) to confirm a diagnosis of hypertension before treatment initiation after initial office screening is recommended by the United States Preventive Services Task Force and 2017 American College of Cardiology and American Heart Association (ACC/AHA) BP guidelines.^1,2^ One tool that may be used to help identify those in need of confirmatory BP monitoring is the Predicting Out-of-Office BP (PROOF-BP) algorithm, which uses office BP measurements and clinical characteristics to predict a patient’s out-of-office BP.^3^ Though many providers report recommending out-of-office BP monitoring to patients, the baseline frequency of its use for specific indications, such as confirming a diagnosis of hypertension, is not known.^4^ Further, barriers relevant to the accessibility and affordability of out-of-office BP monitoring have led to concerns that there may be disparities in the uptake of hypertension screening recommendations.^5^ This analysis examined how historical use of HBPM aligns with current out-of-office BP monitoring recommendations for hypertensive US adults without a previous hypertension diagnosis, and how HBPM use varies by patient characteristics.

## Methods

Adults aged ≥20 years without a diagnosis of hypertension or antihypertensive medication use and a high office BP (≥130/80 mm Hg) who participated in the National Health and Nutrition Examination Survey 2009 to 2014 cycles were identified (n=7185). Included participants had complete data needed to apply the ACC/AHA BP guideline criteria and PROOF-BP algorithm (ie, age, sex, at least three office systolic blood pressure and diastolic blood pressure readings, body mass index, and history of cardiovascular disease).^3^ Participants who reported self-initiated or physician-recommended HBPM were categorized as having used or been told to use HBPM. Participants were categorized as having met criteria to undergo out-of-office BP monitoring according to the 2017 ACC/AHA recommendations if they had a mean office systolic blood pressure/diastolic blood pressure 130 to 159/<100 mm Hg and according to PROOF-BP if they had a predicted out-of-office BP 120 to 134/75 to 84 mm Hg. The age-adjusted proportion of individuals that would meet criteria for out-of-office BP monitoring who reported using or being told to use HBPM was examined overall and was compared by race/ethnicity (ie, non-Hispanic White, non-Hispanic Black, Hispanic, and other), sex, health insurance status, and source of routine health care. All analyses were performed using SAS (version 3.8; Cary, NC) and were weighted to be representative of the 2013 to 2014 US adult population.

## Results

An estimated 31.4 million US adults did not have diagnosed hypertension, were not taking antihypertensive medications and had an office BP ≥130/80 mm Hg. Of the 95.3% (29.3 million) who would have met criteria to undergo out-of-office BP monitoring by the ACC/AHA guidelines, only 3.6% (1.1 million) were told to use HBPM, and 15.7% (4.7 million) had used HBPM (Figure). There were no differences in HBPM use by race/ethnicity, sex, health insurance status, or source of routine healthcare. Though the PROOF-BP algorithm would have recommended fewer individuals for out-of-office BP monitoring (61.9%, 19.5 million), the age-adjusted proportion who were told to use (2.6%, 0.5 million) or used (13.8%, 2.7 million) HBPM was similar overall, and differences were not identified by any of the patient characteristics examined (Table).

**Table.**
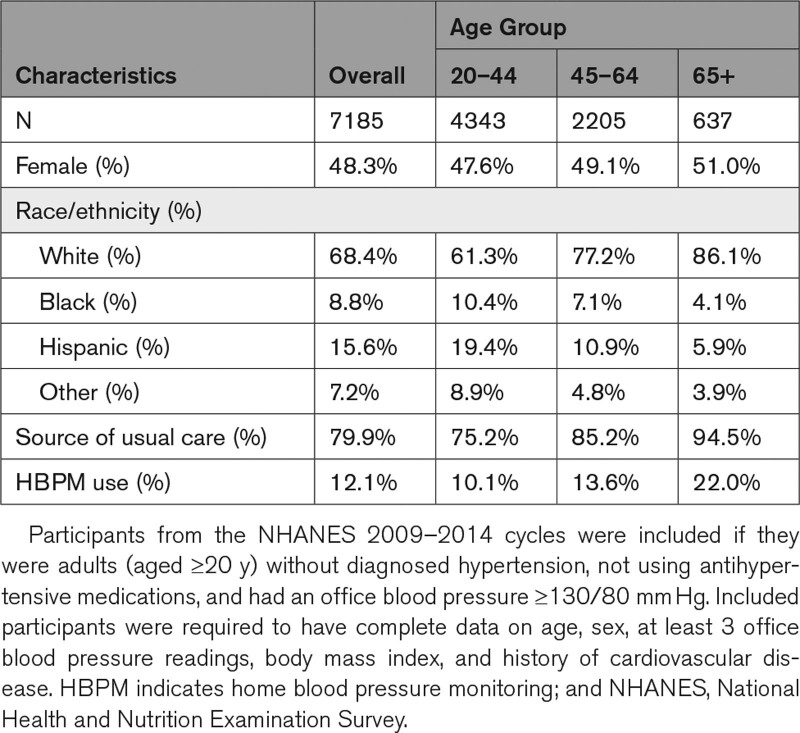
Characteristics and HBPM Use Among Included Participants From NHANES 2009–2014

**Figure.**
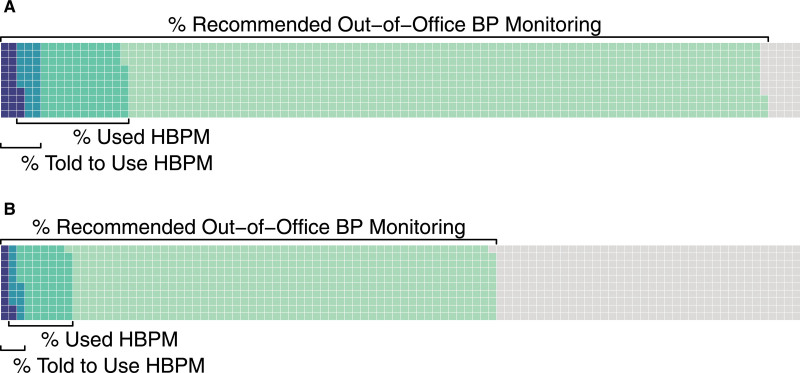
**Out-of-office blood pressure (BP) monitoring recommendations and self-reported use and physician-recommended use of home blood pressure monitoring (HBPM) in National Health and Nutrition Examination Survey (NHANES) 2009–2014.** The figure shows US adults (age ≥20 y) from the NHANES 2009–2014 cycles without diagnosed hypertension, not using antihypertensive medications, and an office blood pressure (BP) ≥130/80 mm Hg (n=7185). **A**, the proportion who would meet criteria to undergo out-of-office BP measurement by the 2017 American College of Cardiology and American Heart Association (ACC/AHA) BP guidelines and the age-adjusted proportion of those who either used or were told to use HBPM by a healthcare provider. **B**, the proportion who would meet criteria to undergo out-of-office BP measurement by the Predicting Out-of-Office Blood Pressure (PROOF-BP) algorithm and the age-adjusted proportion of those who either used or were told to use HBPM by a healthcare provider.

## Discussion

There is a substantial gap between baseline out-of-office BP thresholds for recommended out-of-office BP monitoring and recent HBPM use and recommended use among US adults without a diagnosis of hypertension and a high office BP (≥130/80 mm Hg). Among those with a high office BP who would now meet criteria for out-of-office BP monitoring according to ACC/AHA guidelines or the alternative PROOF-BP strategy, 24.9 million and 16.7 million, respectively, had not used HBPM to confirm hypertension. Evidence-based recommendations from the 2021 United States Preventive Services Task Force report and 2017 ACC/AHA guideline stress the need for confirmatory out-of-office BP measurement before the initiation of antihypertensive medications. To meet these recommendations, HBPM use must dramatically increase, and will require removing systemic barriers to use, including insurance coverage for home BP devices and reimbursement for patient training in home BP use.^4^ The use of a telemonitoring system may improve ease of HBPM use for both physicians and patients, but also introduces additional cost and logistic considerations. Although we did not observe statistically significant differences by race/ethnicity or other patient characteristics, health systems should ensure that implementation plans are equitable and close rather than widen racial/ethnic and geographic hypertension disparities.^5^ These findings are limited in that National Health and Nutrition Examination Survey had office BP from a single visit and were collected before recent screening recommendations; thus, our analyses do not reflect the impact of these guidelines on clinician or patient uptake of HBPM for hypertension diagnosis. Additionally, the impact of the COVID-19 pandemic, which accelerated adoption of remote patient management, on current HBPM use for hypertension diagnosis is unknown. Further, our analyses may underestimate out-of-office BP monitoring as ambulatory BP monitoring data are not available in National Health and Nutrition Examination Survey. However, these data do demonstrate the immense number of individuals who are eligible for out-of-office BP monitoring as part of an evidence-based screening algorithm and quantify an unmet opportunity for clinicians and health systems to improve the quality of hypertension screening.

## Article Information

### Sources of Funding

Dr Bellows receives support through K01 HL140170 from the National Heart, Lung, and Blood Institute, Bethesda, MD. Dr Sheppard receives support through the Wellcome Trust/Royal Society via a Sir Henry Dale Fellowship (ref: 211182/Z/18/Z), National Institute for Health Research (NIHR) School for Primary Care Research, the NIHR Oxford Biomedical Research Centre at Oxford Health NHS Foundation Trust, British Heart Foundation and Stroke Association. Dr Kronish receives funding support from AHRQ (R01 HS024262) and NHLBI (R01 152699). Dr Shimbo receives support through K24HL125704 from the NHLBI. Dr Schwartz receives support from the National Heart, Lung, and Blood Institute (NHLBI) National Institute of Aging, National Institute on Drug Abuse, and the Eunice Kennedy Shriver National Institute of Child Health and Human Development. Dr McManus is a NIHR Senior Investigator and receives support from the NIHR Thames Valley ARC and NIHR School for Primary Care Research. Dr Moran receives support through R01 HL130500-01A1 and R01 HL139837 from the National Heart, Lung, and Blood Institute, Bethesda, MD.

### Disclosures

None.
